# A snap-shot of a diarrheal epidemic in Dhaka due to enterotoxigenic *Escherichia coli* and *Vibrio cholerae* O1 in 2022

**DOI:** 10.3389/fpubh.2023.1132927

**Published:** 2023-04-14

**Authors:** Imam Tauheed, Tasnuva Ahmed, Afroza Akter, Md Golam Firoj, Faisal Ahmmed, Sadia Isfat Ara Rahman, Mokibul Hassan Afrad, Md Nazmul Islam, Aninda Rahman, Ashraful Islam Khan, Baharul Alam, Taufiqur Rahman Bhuiyan, Fahima Chowdhury, Firdausi Qadri

**Affiliations:** ^1^International Centre for Diarrhoeal Disease Research, Bangladesh (icddr,b), Dhaka, Bangladesh; ^2^Directorate General of Health Services, Dhaka, Bangladesh

**Keywords:** ETEC, cholera, co-infection, enterotoxin, colonization factor, seasonal peak, Bangladesh

## Abstract

**Background:**

Enterotoxigenic *Escherichia coli* (ETEC) and *Vibrio cholerae* O1 are most common bacterial causes of diarrheal diseases in Bangladesh. This analysis projected distribution of ETEC and *V. cholerae* O1 among diarrheal patients of icddr,b, Dhaka hospital in two diarrheal peaks of 2022.

**Methodology:**

Under the 2% systematic surveillance system, stool samples collected from diarrheal patients of icddr,b hospital were cultured and diagnostic testing was done for ETEC and *V. cholerae* O1. Comparison of positive cases was done between first peak (March–April) and second peak (October–November) in 2022.

**Results:**

A total of 2,937 stool specimens were tested of which 12% were ETEC and 20% were *V. cholerae* O1. About 40% of the severe dehydration cases were infected with *V. cholerae* O1. Predominant ETEC enterotoxin type was ‘LT/ST’ (41%). The LT enterotoxin significantly increased from 13% to 28% in the second peak (*p* = 0.015). The predominant colonization factors (CFs) on ETEC were CS5 + CS6 (23%), followed by CS6 (15%). CF-positive isolates was significantly higher in the second peak (36%) than in the first peak (22%) (*p* = 0.043). Total 14% cases were co-infected with ETEC and *V. cholerae* O1. Significant differences in the distribution of enterotoxin types were observed (*p* = 0.029) among the co-infection cases.

**Conclusion:**

Changing patterns of enterotoxin and CFs observed in ETEC pathogens should be taken into consideration for ETEC vaccine development. Considering cholera and ETEC biannual trends in causing diarrheal epidemics and outbreaks, emphasizes the need for thoughts on combination vaccine strategies for preventing acute watery diarrhea due to the two major bacterial pathogens.

## Introduction

Diarrheal diseases are one of the major public health problems in developing countries and around 1.5 million deaths occur annually (1). It is consider as the second-highest cause of death among under five years children and it also causes 525,000 deaths in children every year (2). Enterotoxigenic *Escherichia coli* (ETEC) and *Vibrio cholerae* O1 diarrhea together may account for nearly 50% of the diarrheal cases occurring annually in Bangladesh (3). Incidence and severity of the diarrheal diseases causing by these two organisms vary across different age groups. Both ETEC and *V. cholerae* O1 diarrhea can lead to severe dehydration, in adults and children and can contribute to significant morbidity and mortality in resource-poor settings (4). These two bacterial pathogens have similar modes of transmission and can cause community-based infections, as well as hospitalization (5, 6). Both pathogens are endemic with a biannual seasonal peak between March to May (spring/pre-monsoon) and September to November (post-monsoon) (7). The upsurge of ETEC diarrhea leads to the predominance of *V. cholerae* diarrheal epidemics in a few weeks in the spring peak (7).

While pathogenesis of cholera is caused by the excessive production of *V. cholerae* enterotoxin known as cholera toxin (CT) colonizing the small intestine, ETEC diarrhea is also caused by colonization of the intestine and producing heat labile (LT) enterotoxin and heat stable (ST) enterotoxin or combination of both (LT/ST) (8). ST from ETEC strains pathogenic to humans consists of two variants, STh and STp (originated from human and porcine respectively). The LTs of *E. coli* are very similar to CT expressed by *V. cholerae* O1 in its structure and function (8, 9). ETEC expresses a range of plasmid encoded molecules known as colonization factors (CFs) which facilitates intestinal colonization by first adhering to the proximal small intestine enterocytes and then expressing enterotoxins to produce secretory diarrhea (10, 11). More than 25 different CFs are known to be present on ETEC diarrhea in human including CFA/I and CS1–CS22 (10, 12, 13).

In the beginning of March 2022, the capital city Dhaka of Bangladesh and the adjoining areas faced a sudden rise in diarrheal cases. The icddr,b (International Centre for Diarrhoeal Disease Research, Bangladesh) Dhaka hospital is one of the largest hospitals in the world for management of diarrheal patients, where the average patient number is around 160,000 per year (14). In the Dhaka hospital received around 183,175 diarrheal patients between January to November 2022. The icddr,b hospital surged with very high numbers of diarrheal patients between March to April (first peak), and then again between October and November 2022 (second peak). In the first peak, the hospital treated around 62,000 cases which sharply increased during the second week of March 2022. Dhaka hospital treated more than 1,300 patients a day during the first week of April which gradually went down to the pre-epidemic level over the month of May 2022. The peak surge lasted for 9 days. A second surge of diarrhea was observed in October 2022. However, the burden of diarrheal patients was not as high as seen during first peak. This time the number of patients started to increase gradually from the second week of October and peaked in the last week of the month when the patient count went above 1,200 per day and the peak surge lasted for 5 days. In this brief report, we aim to illustrate the trend of ETEC and *V. cholerae* O1 during the diarrheal epidemic periods in 2022 and explore the distribution of colonization factors and ETEC toxin types during the recent outbreaks.

## Methods

### Surveillance for enteric pathogens

The ‘Diarrheal Disease Surveillance System (DDSS)’ of icddr,b Dhaka hospital (2% systematic surveillance), systematically collects data on socioeconomic information, nutritional status, disease severity, clinical management, and disease outcome for every 50^th^ patients coming from all over Dhaka city around the year as well as from other parts of the country for treatment (4). Stool samples were collected from these diarrheal patients for culture to identify the enteric pathogens from January to November (1st week) 2022.

### Microbiological testing for detection of *Vibrio cholerae* and ETEC

*Vibrio cholerae* and ETEC were cultured from stool samples by standard microbiological, conventional serotyping and molecular methods in the Mucosal Immunology and Vaccinology Unit at icddr,b. For the isolation of *V. cholerae* O1, stool samples were cultured in taurocholate- tellurite- gelatin agar (TTGA) plates and incubated overnight at 37°C. By using the specific monoclonal antibodies, we detect the *V. cholerae* O1 Ogawa and Inaba serotypes. The specimens enriched in alkaline peptone water, incubated for additional 18–24 h and then cultured as mentioned above. For detection of ETEC, fecal samples were plated on MacConkey agar and then overnight incubation at 37°C was performed. Six lactose fermenting, individual colonies from MacConkey agar plates were tested by multiplex PCR for the presence of LT and ST (STh, STp) of ETEC toxin (10, 12).

### Identification of ETEC colonization factors

For ETEC positive samples, the colony was plated on to colonization factor antigen (CFA) agar with and without bile salts and was tested for the expression of different CFs (e.g., CFA/I, CS1, CS2, CS3, CS4, CS5, CS6, CS7, CS8, CS12, CS14 and CS17) using CF specific monoclonal antibodies by dot-blot immunoassay. The isolates were also cultured on Trypticase Soy Agar (TSA) plates and tested for CS21 (10, 12).

### Statistical analysis

Descriptive statistics for all the demographic and clinico-pathological characteristics were presented as the frequencies and percentages. Pearson’s Chi-square test has been used to measure the associations with factors contributing to ETEC cholera co-infection. We used proportion test to compare the proportion and rates of enterotoxin types and CFs of ETEC as well as distribution of ETEC and *V. cholerae* among different age groups in the two peaks. All the statistical tests have been conducted at 5% level of significance. Data were analyzed by using R Statistical software. For visualization graphs and plots were created by MS Excel 2007.

### Ethical statement

The 2% systematic surveillance of ‘DDSS’ at icddr,b Dhaka hospital is an ongoing process that has been approved by the icddr,b ‘Institutional Review Board (IRB)’. Verbal consent was taken from each of the patients or from the guardians or caregivers of the patients following hospitalization, assuring them of the confidentiality of information, and taking permission to use the data aimed at analysis for the results. All stored information is used for conducting research and publications (12, 15).

## Results

### Biannual seasonal peak of ETEC and *Vibrio cholerae* O1 in 2022

**Figure 1:**
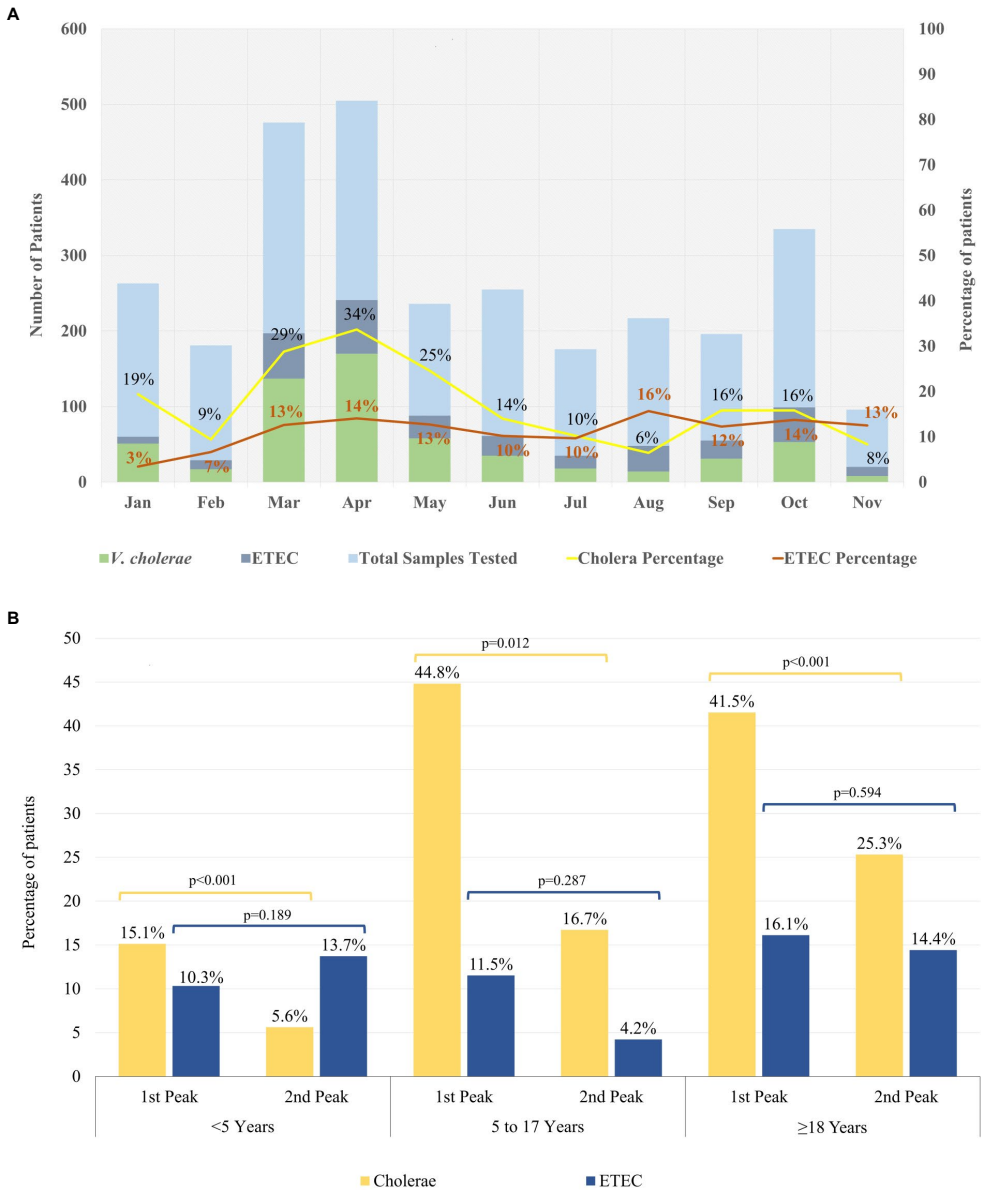
Distribution of ETEC and Cholera in 2022. **(A)** Month wise trend of ETEC and Cholera in 2022. **(B)** Age wise distribution of ETEC and Cholera cases during the biannual peaks in 2022. Chi square test at 5% level of significance was done to identify significant differences between the peaks within the different age groups.

We tested 2,937 samples of diarrheal patients from 2% systemic surveillance, where 52% were below 5 years of age, 7% were 5 to 17 years of age and 41% were above 18 years of age (Table 1). ETEC was positive in 341 (12%) cases and 592 (20%) were positive for *V. cholerae* O1. Figure 1A shows that the proportion of ETEC and cholera cases with the overall diarrheal patients attending the hospital in 2022. In total, 34% of diarrheal patients suffered from severe dehydration of whom 40% were infected with *V. cholerae* O1 and 15% with ETEC (Table 1).

**Table 1:** Distribution of demographic and clinical characteristics of *V. cholerae* and ETEC according to peak (First peak – March to April; Second peak – October to November).

Parameters	Annual*			First peak			Second peak		
	All *n* (%)	ETEC *n* (%)	Cholera *n* (%)	All *n* (%)	ETEC *n* (%)	Cholera *n* (%)	All *n* (%)	ETEC *n* (%)	Cholera *n* (%)
	2,937	341 (11.6)	592 (20.1)	981 (33.4)	131 (13.4)	307 (31.2)	431 (14.7)	58 (13.4)	61 (14.1)
Age group, *n* (%)									
<5 years	1,524 (51.9)	141 (9.3)	118 (7.7)	390 (39.8)	40 (10.3)	59 (15.1)	233 (54.1)	32 (13.7)	13 (5.6)
5–17 years	203 (6.9)	17 (8.4)	72 (35.5)	87 (8.9)	10 (11.5)	39 (44.8)	24 (5.6)	1 (4.2)	4 (16.7)
≥18 years	1,210 (41.2)	183 (15.1)	402 (33.2)	504 (51.4)	81 (16.1)	209 (41.5)	174 (40.4)	25 (14.4)	44 (25.3)
Dehydration status, *n* (%)									
None	1,360 (46.3)	132 (9.7)	91 (6.7)	321 (32.7)	32 (10.0)	42 (13.1)	207 (48.1)	29 (14.0)	10 (4.8)
Some	581 (19.8)	59 (10.2)	107 (18.4)	169 (17.2)	16 (9.5)	52 (30.8)	73 (17.0)	8 (11.0)	9 (12.3)
Severe	995 (33.9)	150 (15.1)	394 (39.6)	491 (50.1)	83 (16.9)	213 (43.4)	150 (34.9)	21 (14.0)	42 (28.0)

* 113 cases were co-infected with ETEC and *V. cholerae*.

During, the first seasonal peak (March–April) of diarrheal cases, we cultured 981 samples from the DDSS of which 307 (31%) were positive for *V. cholerae* O1 and 131 (13%) for ETEC (Table 1). During this period, the proportion of ETEC was almost similar among all age groups (10% among <5 years of age, 12% were 5–17 years, and 16% were ≥ 18 years old patients), while the proportion of *V. cholerae* O1 was found higher among 5–17 and ≥ 18 years old age groups (45, and 42% respectively) compared to patient aged <5 years of age (15%). Among the total severe dehydration (n = 491) cases, 17% of cases were due to ETEC diarrhea whereas 43% were due to *V. cholerae* O1.

In the second seasonal peak, total 431 specimens were tested and a similar distribution of *V. cholerae* O1 and ETEC (14% vs. 13%) was observed. When we stratified the age groups, 14% of ETEC were found among <5 years and ≥ 18 years age group each while only 4% found among 5–17 years of age. *V. cholerae* O1 was isolated 6% among <5 years, 17% in 5–17 years of age and 25% was found in ≥18 years of age (Figure 1B; Table 1). Among the 35% severely dehydrated cases (*n* = 150), 14% diarrheal cases were due to ETEC and 28% were due to *V. cholerae* O1. The rate of cholera decreased significantly from 15 to 6% in the second peak among the <5 years age group (*p* < 0.001) but there were no significant changes in the ETEC rate (Figure 1B), although ETEC diarrhea cases were higher than cholera cases in the second peak among the <5 years old children.

The proportion of cholera cases among the overall diarrheal patients attending the hospital was higher in the first peak than the second peak (Figure 1A). During 2022, both serotypes of *V. cholerae* O1 Inaba (54%) and Ogawa (46%) were identified. However, during the first peak *V. cholerae* O1 Inaba was the predominant serotype (75%) while during the second peak 98% of the *V. cholerae* O1 isolates were Ogawa serotype (Supplementary Figure S1).

### Distribution of ETEC enterotoxin and colonization factors

**Figure 2:**
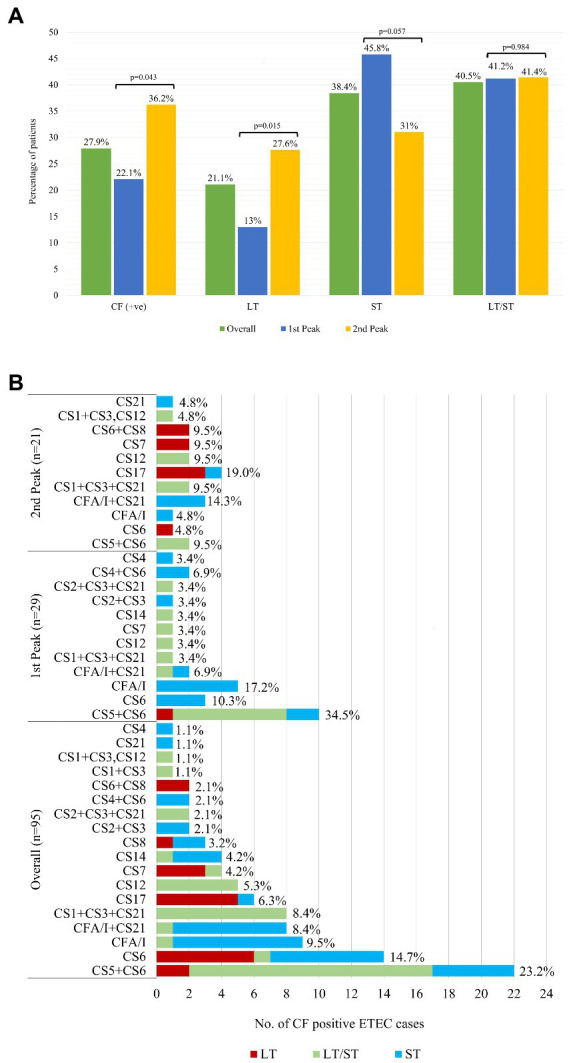
Distribution of ETEC enterotoxins (LT, ST, LT/ST) and colonization factors (CFs). **(A)** Overall Distribution of ETEC enterotoxins (LT, ST, LT/ST) and colonization factors (CFs) along with the peaks. Comparison of the enterotoxin type (LT, ST and LT/ST) and colonization factors (CFs) isolates between the first and second peak was done using Chi square test at 5% level of significance. **(B)** Distribution of ETEC Colonization Factors (CFs) as per enterotoxin.

The distribution of ETEC enterotoxin type (LT, ST, and LT/ST) is shown in Figure 2A. Of the 3 different types of ETEC enterotoxin, 41% were LT/ST (78% LT/STh, 20% LT/STp, and the rest 2% LT/STh/STp), 38% ST (76% STh, 23% STp and the rest STH/STp 1%), and 21% LT (Figure 2A; Supplementary Table S1). The overall predominant ETEC enterotoxin type in 2022 was the ‘LT/ST’ toxin type (41%). In the first peak, the distribution of ST and LT/ST were similar (46 and 41%) whereas the ‘LT’ enterotoxin types were only 13% (Figure 2A). During the second peak, LT/ST (41%) was the most prevalent enterotoxin of ETEC diarrhea. The LT toxin type significantly increased from 13 to 28% in the second peak (*p* = 0.015) while ST toxin type decreased to 31% in second peak compared to the first peak (*p* = 0.057). From the ETEC diarrhea cases, 28% of the ETEC isolates were positive for CFs, where 41% (*n* = 39) were of ST only, 39% (*n* = 37) were of LT/ST, and 20% (*n* = 19) were of LT only enterotoxin. A significant increase in the CF positive isolates was observed in the second peak (36%) compared to the first peak (22%; *p* = 0.043; Figure 2A).

The most frequent CF detected was CS5 + CS6 with 22/95 (23%), followed by CS6 with 14/95 (15%), CFA/I with 9/95 (10%), CFA/I + CS21 8/95 (8%) and CS1 + CS3 + CS21 with 8/95 (8%), CS17 and CS12 were 6 and 5%, respectively, (Figure 2B). CS5 + CS6, CS6 were common across all toxin combinations (on ETEC as LT only, ST only and a combination of LT/ST) where CS5 + CS6 was most frequent in LT/ST combination and CS6 was higher in LT only and ST only toxin type. CFA/I and CFA/I + CS21 were found mostly in only ST enterotoxin while CS1 + CS3 + CS21 and CS12 were identified in LT/ST strains (Figure 2B).

### Co-infection with ETEC and *Vibrio cholerae* O1

In total, 14% (*n* = 113) of the cases were co-infected with ETEC and *V. cholerae* O1 of which 56% of the co-infected cases were found during the first seasonal peak and 7% were found in second seasonal peak (Table 2). The most common CF for ETEC pathogen as co-infection was CS5 + CS6.

**Table 2:** Distribution of co-infection among either cholera or ETEC positive patients.

Parameter		Co-infection *n* = 113	Single infection (either cholera or ETEC) *n* = 707	Value of p*
Age	<5 Years	18 (15.9%)	223 (31.5%)	0.003
	5–17 Years	12 (10.6%)	65 (9.2%)	
	≥18 Years	83 (73.5%)	419 (59.3%)	
Severity	None	17 (15.0%)	189 (26.7%)	<0.001
	Some	14 (12.4%)	138 (19.5%)	
	Severe	82 (72.6%)	380 (53.8%)	
Peaks^a^	First Peak	63 (16.8%)	312 (83.2%)	0.026
	Second Peak	8 (7.2%)	103 (92.8%)	
		Co-infection *n* = 113	Single infection (ETEC)	
Toxin type (*N* = 341)	LT	19 (16.8%)	53 (23.2%)	0.029
	ST	37 (32.7%)	94 (41.2%)	
	LT/ST	57 (50.4%)	81 (35.5%)	
CF Result	Positive	12 (10.6%)	83 (36.4%)	<0.001
	Negative	101 (89.4%)	145 (63.6%)	

* Chi square test was performed to measure the association factors contributing to co-infection at 5% level of significance.

a Data shown in No. (Row percentage).

Most (73%) of the co-infection cases were in ≥18 years old age group, compared to <5 and 5–17 years of age (16 and 11%) and is statistically significant (*p* = 0.003). A total of 73% of the co-infected cases suffered from severe dehydration (*p* < 0.001) compared to some and no sign of dehydration. Half of the co-infection cases contributed to “LT/ST” enterotoxin type whereas 33% (*n* = 37) were “ST” and the rest 17% (*n* = 19) were “LT” and the differences were statistically significant (*p* = 0.029; Table 2).

## Discussion

This report describes the distribution of two major bacterial diarrheal pathogens ETEC and *V. cholerae* O1, ETEC toxin profiles along with colonization factors, dual infection and the disease severity of these two pathogens leading two seasonal outbreaks for the year 2022.

The most interesting observation in this analysis revealed that in the first seasonal peak, *V. cholerae* O1 was higher whereas ETEC was gradually increasing but sudden upsurge of *V. cholerae* O1 played an important role in causing the epidemic. Thereafter, the proportionality of cholera cases tended to decline, though the rate of ETEC cases continued to rise and peaked (16%) when cholera cases (6%) dropped dramatically in August 2022 among the tested samples.

In the second peak, the prevalence of both ETEC and *V. cholerae* O1 were similar among all diarrheal cases. Earlier studies demonstrated that cholera infection and disease are characteristically seasonal with one major and one smaller peak annually, which is similar to our observation that the spring peak occurred mainly due to *V. cholerae* O1. Environmental signatures may play an important role for the lower surge of *V. cholerae* in the second peak (7, 15). Another explanation for the lower number of cholera cases in the second peak may be due to a reactive oral cholera vaccination campaign, held between June to August 2022, conducted in five identified hotspot areas of Dhaka city from where the majority of the diarrheal cases seek care at the icddr,b Dhaka hospital (16, 17). Moreover, the severity of dehydration was more prominent in the first peak (50%) which reduced to 35% in the second seasonal peak of the year. This may be due to a lower number of cholera cases presenting in the latter peak (almost half from the first seasonal peak) and increased ETEC cases during the second surge of diarrheal cases.

Interestingly, in our current analysis we found that the both LT/ST and ST enterotoxins were predominant among the ETEC cases and majority of the CF positive isolates were among the LT/ST and ST enterotoxins which was similar to the earlier studies of 1996–1998 and 2002–2004 period (10, 18, 19). However, evidence from 2007 showed that LT phenotype were predominant during that period and that the CF expressing strains were in higher proportion from the LT phenotype alone than ST or LT/ST phenotypes (7, 18).

Our current analysis revealed that CS5 + CS6 and CS6 CFs were the most prevalent isolates while CS1 + CS3, CS21, CS4, and CS1 + CS3 + CS12 were the least prevalent ones which is similar to earlier studies (6, 19). But during the period 2003–2006, CS7, CS17 and CFA/1 and during the 2007–2012, C7, and C17 were also predominant in Bangladesh (3, 7, 12). It seems that ETEC pathogens are undergoing changes in both toxin and CF types. Therefore, these changing phenotypes of ETEC pathogens should be taken under consideration for future ETEC vaccine development to prevent ETEC diarrheal epidemics as they contribute to growth stunting and cognitive impairment among infants and young children (under 5 years of age). Anderson et al. showed in children younger than 5 years, ETEC diarrhea resulted in 3·5 million (0.8–5.4) cases of moderate-to-severe stunting and 44 400 (29,400–59,800) total ETEC deaths in 2015 (20). With the introduction of ETEC vaccines 92,000 [61,200; 129,900] ETEC direct deaths and 21,800 [11.29; 34.75] ETEC- induced stunting deaths from other infectious diseases over the first 10 years can be prevented (21).

The most recent ETEC vaccine under development is *Escherichia coli* (ETEC) vaccine ETVAX with promising immunogenicity data of phase I/II clinical trial (22, 23), consisting of inactivated recombinant *E. coli* strains over-expressing the colonization factors (CFs) CFA/I, CS3, CS5 and CS6 and the heat-labile toxoid LCTB*A.* The common CFs observed from this analysis include CFA/I, CS5, and CS6 which are assumed to be protected by ETVAX however there is no protective efficacy data on this vaccine.

This analysis revealed that around 14% of patients were co-infected by both ETEC and *V. cholerae* O1, which is very similar to earlier studies that mixed pathogens are identifiable in 12% to 26% of individuals with acute watery diarrhea in Bangladesh (3, 10). We observed that co-infection suffered significantly from severe dehydration and that majority of the cases were adults which was comparable with earlier studies in endemic areas of cholera and ETEC diarrhea (3, 6, 24, 25). Only 12 of 113 (11%) were CF positive in the ETEC and *V. cholerae* O1 co-infected cases where the overall CFs positive for all ETEC cases was 28%. In co-infection with *V. cholerae* O1, CS5+CS6 colonization factor of the ETEC pathogen were the dominant CFs, which was also the dominant colonization factor among all the ETEC diarrheal cases.

In this analysis, samples were collected from patients seeking hospital care service, therefore the severity of the cases presented here may be higher than seen in community studies (24). It was also seen that both ETEC and *V. cholerae* O1 can cause severe disease including 9–42% asymptomatic infections in household contacts and in the community (3, 6, 19, 25). Likewise, the practice of taking ORS as well as self-medication or antibiotics at home during diarrhea may be keeping a lot of people at home with mild and moderate dehydration.

Although our data clearly support the predominance of *V. cholerae* O1 and ETEC as the main cause of seasonal diarrheal epidemics, it is important to further explore other diarrheal pathogens contributing to the increase in the number of acute watery diarrheal patients seeking health care services during the associated epidemics. Previous analysis (in 2018) of the 2% surveillance samples from patients which is carried out routinely at icddr,b showed that apart from *V. cholerae* O1 and ETEC, other pathogens such as *Shigella* spp. (~1.6%) and *Campylobacter* spp. (~13%), were also seen during the diarrheal epidemic (15).

Strategies for use of available cholera and ETEC vaccine is needed in the areas before the seasonal peaks to reduce the burden of ETEC and cholera. We see that ETEC diarrhea was responsible for rise in hospitalization of children under 5 years of age which was evident in the second peak between October to November. At present, Dukoral is the only vaccine available which provides protection against diarrheal diseases caused by *V. cholerae* (85%) and ETEC (67%) (26). However, the cost of the Dukoral vaccine is too high and is very difficult to use in emergency setting as it requires a buffer solution to protect its functionality (5). Bangladesh Government is implementing its National Cholera Control Plan (NCCP) to eradicate cholera from the country. An efficient surveillance system and geospatial mapping of identified cholera hotspots is ongoing. Locally produced OCV ‘Cholvax’ has been manufactured and licensed in Bangladesh will minimize the recent demand for OCV when achieve the WHO prequalification. Delivering OCV in different hotspot areas of the country in conjunction with WASH strategies; e.g. effective sewage systems with safe waste disposal and mechanisms to prevent untreated waste from reentering the environment, along with health education and prophylactic antibiotic treatment are essential for controlling of recurrent diarrheal episodes as they are resulting mainly due to inadequate sanitation, poor food hygiene and drinking contaminated water, along with spillage of contaminants into the supply water system and other water reservoirs during the monsoon.

We have moved ahead with preventive and reactive vaccination with oral cholera vaccine in Bangladesh (16, 17), however the need for a ETEC vaccine appears critical and also combination vaccines that can be given for control of cholera and ETEC diarrhea in regions like Bangladesh where both pathogens are responsible for hospitalization in health facilities. The burden of these diseases in the community needs to be addressed urgently especially for young children which can lead to substantial mortality but disproportionately leads to high morbidity.

## Data availability statement

The raw data supporting the conclusions of this article will be made available by the authors, without undue reservation.

## Ethics statement

The studies involving human participants were reviewed and approved by Institutional Review Board (IRB) of icddr,b. Verbal consent was taken from each of the patients or from the guardians or caregivers of the patients of 2% systematic surveillance of ‘DDSS’ following hospitalization, assuring them of the confidentiality of information, and taking permission to use the data aimed at analysis for the results.

## Author contributions

FC, and FQ conceptualized the study. IT, TA, MGF and FA analyzed the data. IT and TA drafted the manuscript. TRB, SIAR, and MHA helped to performed the laboratory work. FQ, FC, TRB, AA, SIAR, MNI, AR, AIK, and BA reviewed the manuscript. All authors contributed to the interpretation of results and critical review and revision of the manuscript and have approved the final version.

## Conflict of interest

The authors declare that the research was conducted in the absence of any commercial or financial relationships that could be construed as a potential conflict of interest.

## Publisher’s note

All claims expressed in this article are solely those of the authors and do not necessarily represent those of their affiliated organizations, or those of the publisher, the editors and the reviewers. Any product that may be evaluated in this article, or claim that may be made by its manufacturer, is not guaranteed or endorsed by the publisher.
