# Supplementary material for: A snap-shot of a diarrheal epidemic in Dhaka due to enterotoxigenic Escherichia coli and Vibrio cholerae O1 in 2022
Source: Front Public Health. 2023 Apr 14;11:1132927. doi: 10.3389/fpubh.2023.1132927 (PMC10140589; doi:10.3389/fpubh.2023.1132927)
Supplement: Supplementary file 1 [file Table_1.docx]

**Supplementary Table 1. Distribution of ETEC enterotoxin LT, ST (STh, STp, and STh/STp), and LT/ST (LT/STh, LT/STp, and LT/STh/STp) along with the peaks in 2022**

| **Parameters** | | **LT** | **STh** | **STp** | **STh/STp** | **LT/STh** | **LT/STp** | **LT/STh/STp** |
| --- | --- | --- | --- | --- | --- | --- | --- | --- |
|  | | **72** | **100** | **30** | **1** | **108** | **28** | **2** |
| Age Group  **(N=341)** | <5 Years | 44 (31.2%) | 36 (25.5%) | 16 (11.3%) | 1 (0.7%) | 23 (16.3%) | 19 (13.5%) | 2 (1.4%) |
|  | 5 to 17 Years | 1 (5.9%) | 10 (58.8%) | 3 (17.6%) | 0 | 3 (17.6%) | 0 | 0 |
|  | ≥18 Years | 27 (14.8%) | 54 (29.5%) | 11 (6.0%) | 0 | 82 (44.8%) | 9 (4.9%) | 0 |
| Severity  **(N=341)** | None | 35 (26.5%) | 41 (31.1%) | 11 (8.3%) | 1 (0.8%) | 27 (20.5%) | 15 (11.4%) | 2 (1.5%) |
|  | Some | 11 (18.6%) | 14 (23.7%) | 10 (16.9%) | 0 | 18 (30.5%) | 6 (10.2%) | 0 |
|  | Severe | 26 (17.3%) | 45 (30%) | 9 (6.0%) | 0 | 63 (42.0%) | 7 (4.7%) | 0 |
| Peak | 1st Peak | 17 (13.0%) | 50 (38.2%) | 10 (7.6%) | 0 | 48 (36.6%) | 5 (3.8%) | 1 (0.8%) |
|  | 2nd Peak | 16 (27.6%) | 16 (27.6%) | 2 (3.4%) | 0 | 18 (31.0%) | 5 (8.6%) | 1 (1.7%) |
